# Long-Range Correlation Underlying Childhood Language and Generative Models

**DOI:** 10.3389/fpsyg.2018.01725

**Published:** 2018-09-19

**Authors:** Kumiko Tanaka-Ishii

**Affiliations:** Research Center for Advanced Science and Technology, University of Tokyo, Tokyo, Japan

**Keywords:** long-range correlation, fluctuation analysis, CHILDES, generative models, Simon Model, Pitman-Yor model

## Abstract

Long-range correlation, a property of time series exhibiting relevant statistical dependence between two distant subsequences, is mainly studied in the statistical physics domain and has been reported to exist in natural language. By using a state-of-the-art method for such analysis, long-range correlation is first shown to occur in long CHILDES data sets. To understand why, generative stochastic models of language, originally proposed in the cognitive scientific domain, are investigated. Among representative models, the Simon model is found to exhibit surprisingly good long-range correlation, but *not* the Pitman-Yor model. Because the Simon model is known not to correctly reflect the vocabulary growth of natural languages, a simple new model is devised as a conjunct of the Simon and Pitman-Yor models, such that long-range correlation holds with a correct vocabulary growth rate. The investigation overall suggests that uniform sampling is one cause of long-range correlation and could thus have some relation with actual linguistic processes.

## 1. Introduction

State-of-the-art generative mathematical models of language include the Simon and Pitman-Yor models and their extensions (Pitman, 2006; Chater and Oaksford, 2008; Lee and Wagenmakers, 2014). One of the first studies of these models included application to data in developmental psychology (Goldwater et al., 2009, 2011). These models have been not only successful in modeling language development from a cognitive perspective but also applicable in natural language engineering (Teh, 2006). They have been adopted primarily because the rank-frequency distribution of words in natural language follows a power law. Advances in studies on the statistical nature of language have revealed other characteristics besides Zipf's law. For example, Heaps' law describes how the growth of vocabulary forms a power law with respect to the total size (Guiraud, 1954; Herdan, 1964; Heaps, 1978); the Pitman-Yor model follows this principle well.

In this paper, another power law underlying the autocorrelation function of natural language is considered. Called long-range correlation, it captures a qualitatively different characteristic of language. As described in detail in the following section, long-range correlation is a property of time series that has mainly been studied in the statistical physics domain for application to natural and financial phenomena, including natural language. When a text has long-range correlation, there exists a (yet unknown) structure underlying the arrangements of words. One rough, intuitive way to understand this is by the tendency of rare words to cluster. The phenomenon is actually more complex, however, as it has been reported to occur at a long scale. Because the methods used to investigate this phenomenon measure the similarity between two long subsequences within a sequence, long-range correlation suggests some underlying self-similarity. In other words, it is not only the case that rare words cluster, but more precisely, that words at all different rarity levels tend to cluster.

Verification of the universality of long-range correlation in language is an ongoing topic of study and has been reported across domains. In linguistics, it has been shown through hand counting how rare words cluster in the *Iliad* (van Emde Boas, 2004). Computational methods from the statistical physics domain have given multiple indications of the existence of long-range memory in literary texts (Ebeling and Pöschel, 1994; Altmann et al., 2009; Tanaka-Ishii and Bunde, 2016). Moreover, long-range correlation has been reported to occur across multiple texts (Serrano et al., 2009) and also in news chats (Altmann et al., 2009). In recent years, using the methods proposed in the statistical physics domain, analysis of long-range correlation has been reported. For example, Bedia et al. (2014) shows how social interaction is long-range correlated by using detrended fluctuation analysis, and Ruiz et al. (2014) shows how skilled piano play also is long-range correlated and how it is related to auditory feedback.

In this article, first, long-range correlation for long sets of CHILDES data is reported. The fact that power-law behavior exists in early childhood language is surprising, because children's linguistic utterances seem undeveloped, lacking vocabulary and proper structure, and full of grammatical errors. Given the power law indicated by the autocorrelation function, there must be an innate mechanism for the human language faculty.

To explore the source of this mechanism, the article investigates how this autocorrelative nature is present in generative models, which are one kind of state-of-the-art model originating in psychology (Simon, 1955). The sequences generated by a Pitman-Yor model (Pitman, 2006) are not long-range correlated, which raises a question of the validity of Pitman-Yor models in scientific language studies. In contrast, the Simon model (Simon, 1955), the simplest model commonly adopted in complex systems studies, has strong long-range correlation. Given how the Simon model works, this suggests that one cause of the autocorrelative nature lies in uniform sampling from the past sequence along with introduction of new words from time to time. Because the Simon model has a drawback with respect to vocabulary growth, a simple conjunct model is constructed so as to produce both long-range correlation and correct vocabulary growth. In conclusion, the article discusses the relation between uniform sampling and linguistic procedures.

## 2. Quantification of long-range correlation

**Figure 1:**
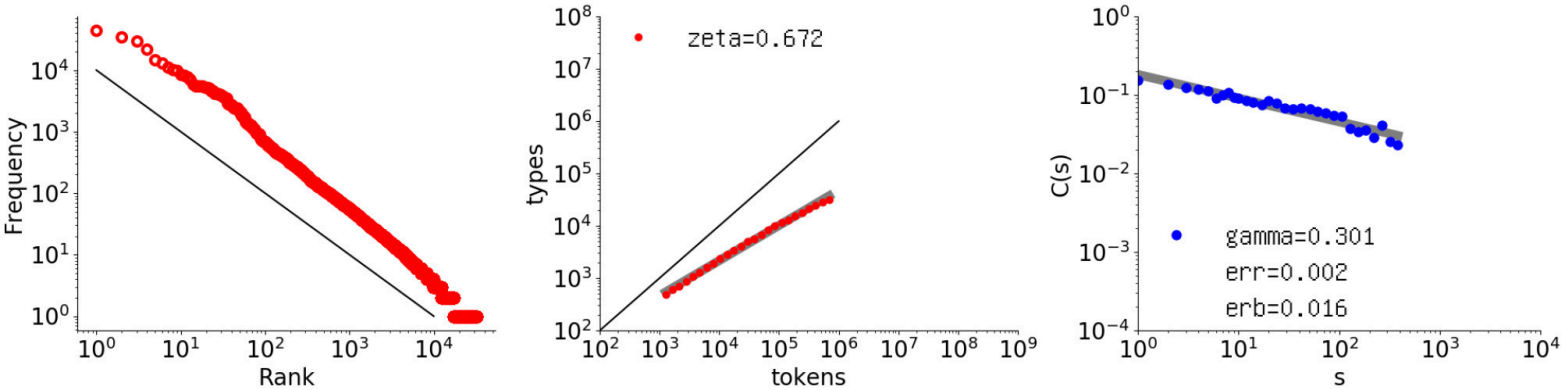
Log-log plots of the rank-frequency distribution, type-token relation, and autocorrelation function for *Les Misérables* (by V. Hugo (French), number of words 691407). **Left** Rank-frequency distribution, where the x-axis indicates the rank, and the y-axis indicates the frequency. The red points represent the actual data, and the black line indicates a slope of ξ = 1.0. **Middle** Type-token relation, where the x-axis indicates the text size in words, and the y-axis indicates the vocabulary size. The set of red points represents the actual data, along with its fit line in light gray, and the black line indicates a slope of ζ = 1.0. The fitted exponent is shown in the upper left corner. **Right** Autocorrelation function applied to intervals, where the x-axis indicates the offset *s*, and the y-axis indicates the value of the autocorrelation function *C*(*s*) for an interval sequence consisting of one-sixteenth of all the words. The blue points represent the actual data, the thick gray line is the fitted power law, and the slope γ is shown in the lower left corner, with the residual denoted as *err* and the error of the slope as *erb*.

The focus of this paper is the power law observed for the autocorrelation function when applied to natural language. As an example, the rightmost graph in Figure 1 shows the autocorrelation function applied to the text of *Les Misérables*. The points are aligned linearly in a log-log plot, so they follow a power law. The correlation is *long*, in contrast to short-range correlation, in which the points drop much earlier in an exponential way.

There is a history of nearly 25 years of great effort to quantify this long-range correlation underlying text. Because all the existing analysis methods for quantifying long-range memory—i.e., the autocorrelation function that is defined and used later in this section, fluctuation analysis (Kantelhardt et al., 2001; Kantelhardt, 2002), and rescaled range (R/S) analysis (Hurst, 1951)—apply *only* to numerical data, much effort has focused on the question of how best to apply these methods to linguistic (thus, non-numerical) sequences. Previous studies applied one of these methods to a binary sequence based on a certain target word (Ebeling and Pöschel, 1994), a word sequence transformed into corresponding frequency ranks (Montemurro and Pury, 2002), and so on. State-of-the-art approaches use the concept of intervals (Altmann et al., 2009; Tanaka-Ishii and Bunde, 2016), with which a numerical sequence is derived naturally from a linguistic sequence. Note that this transformation into an interval sequence is not arbitrary as compared with other transformations, such as the one into a rank sequence. An approach using only interval sequences, however, suffers from the low-frequency problem of rare words, and clear properties cannot be quantified even if they exist. Here, instead, the analysis uses the method proposed in Tanaka-Ishii and Bunde (2016), which conducts interval analysis for a set of rare words treated as extreme events.

This method of interval analysis inspired by extreme value analysis was established within the statistical physics domain, originally for analyzing extreme events with numerical data, such as devastating earthquakes. Analysis schemes using intervals between such rare events always consider rarer events above a threshold (corresponding here to *N*), to tackle the low-frequency problem. Various complex systems are known to exhibit long-range correlation (or long-range memory), as reported in the natural sciences and finance (Turcotte, 1997; Corral, 2004, 2005; Bunde et al., 2005; Santhanam and Kantz, 2005; Bogachev et al., 2007; Yamasaki et al., 2007; Blender et al., 2015). By assuming that rare words in a language sequence should correspond to extreme events, the analysis scheme was hence developed as reported in Tanaka-Ishii and Bunde (2016). That work showed how 10 single-author texts exhibit long-range correlation. Thus, among multiple reports so far, there is abundant evidence that language has long-range correlation in its word arrangement.

A self-contained summary of the analysis scheme is provided here, and a detailed argument for the method is found in Tanaka-Ishii and Bunde (2016). The method basically uses the autocorrelation function to quantify the long-range correlation. Given a numerical sequence *R* = *r*_1_, *r*_2_, …, *r*_*M*_, of length *M*, let the mean and standard deviation be μ and σ, respectively. Consider the following autocorrelation function:

(1) $$C(s)=\frac{1}{(M-s)\sigma^{2}}\sum_{i=1}^{M-s}\left(r_{i}-\mu )(r_{i+s}-\mu \right).$$

This is a fundamental function to measure the correlation, the similarity of two subsequences separated by distance *s*: it calculates the statistical covariance between the original sequence and a subsequence starting from the *s*th offset element, standardized by the original variance of σ^2^. For every *s*, the value ranges between −1.0 and 1.0, with *C*(0) = 1.0 by definition. For a simple random sequence, such as a random binary sequence, the function gives small values fluctuating around zero for any *s*, as the sequence has no correlation with itself. The sequence is judged to be long-range correlated when *C*(*s*) decays by a power law, as denoted in the following:

(2) $$\begin{array}{r} C\left(s\right)\propto s^{-\gamma }. \end{array}$$

The particularity of the autocorrelation lies in its long-range nature: two subsequences existing in a sequence remain similar even if *s* becomes fairly large. Short-term memory, which is the exponential decay of correlation, shows how the target relies only on local arrangements, in a Markovian way. In contrast, the long-range correlation is considered important precisely because such correlation lasts long. For a natural language sequence, too, we want to calculate *C*(*s*) and verify whether it exhibits power-law decay. The essential problem lies in the fact that a language sequence is not numerical and thus must be transformed into some numerical sequence.

**Figure 2:**
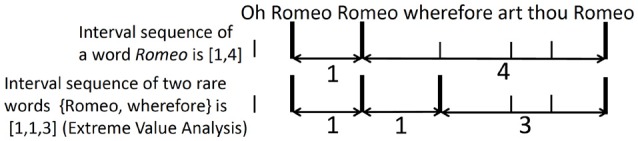
A toy example of interval analysis and extreme value analysis.

The method of Tanaka-Ishii and Bunde (2016) transforms a word sequence into a numerical sequence by using intervals of rare words. The following example demonstrates how this is done. Consider the target *Romeo* in the sequence “Oh Romeo Romeo wherefore art thou Romeo,” shown in Figure 2. *Romeo*, indicated by the thick vertical bar, has a one-word interval between its first and second occurrences, and the third *Romeo* occurs as the fourth word after the second *Romeo*. This gives the numerical sequence [1, 4] for this clause and the target word *Romeo*. The target does not have to be one word but can be any element in a set of words. Suppose that the target consisted of two words, the two rarest words in this clause: *Romeo*, and *wherefore*. Then, the interval sequence would be [1,1,3], because *wherefore* occurs right after the second *Romeo*, and the third *Romeo* occurs as the third word after *wherefore*. As rare words occur only in small numbers, consideration of multiple rare words serves to quantify their behavior as an accumulated tendency.

**Figure 3:**
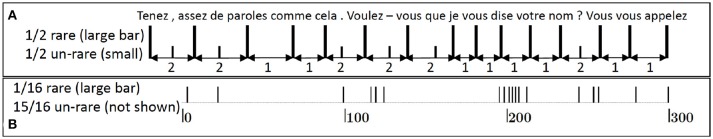
Illustrations of **(A)** the procedure to acquire an interval sequence and **(B)** how rare words are clustered in a part of *Les Misérables*.

Figure 3 illustrates the analysis scheme for a longer sequence. The Figure 3A shows an example from *Les Misérables* in which half of the words in the text are considered rare (large bars), and the other half are considered common (small bars). By using the large bars, the text panel is transformed into an interval sequence shown at the bottom as [2,2,1,…], similarly to the Romeo example in Figure 2. In the Figure 3B, one sixteenth of all the words, instead of half, are considered rare. The locations of only the large bars are shown for a passage of 300 words starting from the 31096th word in *Les Misérables*: the bars appear in a clustered manner. Such clustering of events is a typical characteristic of a long-range memory process. When events are clustered, then, the waiting times between events are clustered, too. Therefore, the autocorrelation function must exhibit correlation at a long scale.

As a summary, the overall procedure is described as follows. Given a numerical sequence of length *M*, the number of intervals for one *N*th of (rare) words is *M*_*N*_ ≡ *M*/*N* − 1^1^. For the resulting interval sequence *R*_*N*_ = *r*_1_, *r*_2_, …, *r*_*M*_*N*__ (where *r*_*i*_ is the interval between the *i*th and *i* + 1-th occurrence of a word in *M*_*N*_ words), let the mean and standard deviation be μ_*N*_ and σ_*N*_, respectively. Then the autocorrelation function is calculated for this *R*_*N*_, with *M*_*N*_, μ_*N*_, and σ_*N*_ replacing *M*, μ, and σ, respectively, in formula (1).

For literary texts, *C*(*s*) takes positive values forming a power law (Tanaka-Ishii and Bunde, 2016). The blue points in the rightmost graph in Figure 1 represent the actual *C*(*s*) values in a log-log plot for a sequence of *Les Misérables* in its entirety^2^. The thick gray line represents the fitted power-law function, which shows that this degree of clustering decays by a power law with exponent γ = 0.301 with the slope error *erb* = 0.0159 (the standard deviation of γ), and a fit error (residual) of *err* = 0.00158 per point^3^. The points are fitted to a linear function in log-log coordinates by the least-squares method. The points are all positive within the chosen range of *s*.

Following a previous work, the long-range correlation is reconsidered here through CHILDES data and mathematical generative models. In Tanaka-Ishii and Bunde (2016), *N* was varied across 2, 4, 8, 16, 32, 64. For large *N*, the interval sequence became too short for proper analysis, but for small *N*, it included words that occur too frequently. To focus on the main point of the article without having too many parameters, *N* = 16 is used throughout the remainder.

The main contribution of this paper is to discuss generative models in seeking the reason why such long-range correlation exists. Before proceeding, two other, more common power laws are introduced because they are necessary for the later discussion in 4. The leftmost graph in Figure 1 shows the log-log rank-frequency distribution for *Les Misérables*, which demonstrates a power-law relationship between the frequency rank and frequency, i.e., Zipf's law. Given word rank *u* and frequency *F*(*u*) for a word of rank *u*, Zipf's law suggests the following proportionality formula:

(3) $$\begin{array}{r} F\left(u\right)\propto u^{-\xi },\;\xi \approx 1.0. \end{array}$$

As shown here for *Les Misérables*, the plot typically follows formula (3) only approximately. There have been discussions on how to improve the Zipf model by incorporating such bias (Mandelbrot, 1952, 1965; Gerlach and Altmann, 2013; Deng et al., 2014). To the best of the author's knowledge, however, the question of a mathematical model that fully explains the bias is still under debate.

The middle graph in Figure 1 shows the type-token relation based on another power law, usually referred to as Heaps' Law, indicating the growth rate of the vocabulary size with respect to the text length. Given vocabulary size *V*(*m*) for a text of length *m*, Heaps' law is as follows:

(4) $$\begin{array}{r} V\left(m\right)\propto m^{\zeta },\;\zeta <1.0. \end{array}$$

This feature was known even before (Heaps, 1978), as published in Herdan (1964) and Guiraud (1954). In the graph, the black line represents an exponent of 1.0. As seen here, for *Les Misérables*, ζ = 0.672, much smaller than 1.0; indeed, the growth rate for natural language is below 1.0.

## 3. Autocorrelation functions for childhood language

Using the method introduced in the previous section, this section introduces a kind of data that has never been considered in the context of long-range correlation: child-directed speech corpus, CHILDES (MacWhinney, 2000). In contrast to the previous work on single-author texts, these data concern utterances (speech). Furthermore, the data are chronologically ordered, thus showing the development of a child's linguistic capability.

**Figure 4:**
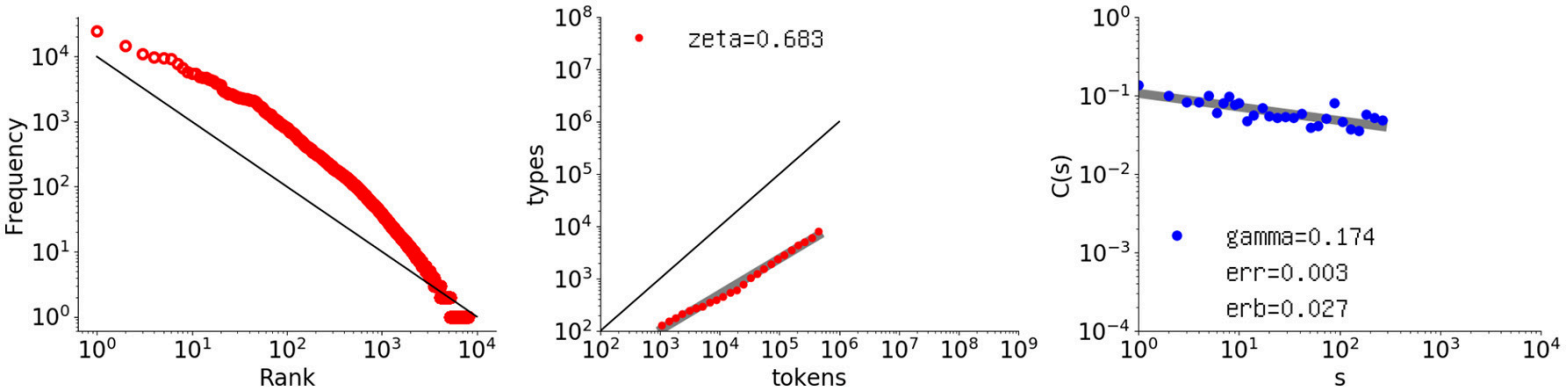
Rank-frequency distribution, type-token relation, and autocorrelation function for the Thomas data set (448771 words). The thick gray lines show the estimates fitting a linear model (second and third graphs), whereas the thin black lines indicate the slopes of –1 and 1, for the first and second graphs, respectively.

The first example is Thomas (in English), which is the longest data set in CHILDES (Lieven et al., 2009) (448771 words). Figure 4 shows the rank-frequency distribution, type-token relation, and autocorrelation function for Thomas' utterances, similarly to Figure 1.

The autocorrelation function (right) has a surprisingly tight power law, thus indicating long-range correlation. Because a child's utterances are linguistically under development, this result is not trivial. The slope is smaller than that of the literary text being γ = 0.174 with slope error *erb* = 0.0273. None of the calculated *C*(*s*) values are negative, and the fit error *err* = 0.00255 per point.

As for the rank-frequency distribution (left), the overall slope is almost 1.0 but the plot has a clear convex tendency, as compared with the black line representing a slope of 1.0. Such convex tendency of the rank-frequency distribution of CHILDES data has been studied elsewhere, from another perspective such as in Baixeries et al. (2013). It suggests that Thomas generated utterances by using more frequent words, especially the top 100 words.

Lastly, the middle graph shows the type-token relation. As compared with *Les Misérables*, the vocabulary growth is less stable and slightly steeper, with an exponent of ζ = 0.683.

**Figure 5:**
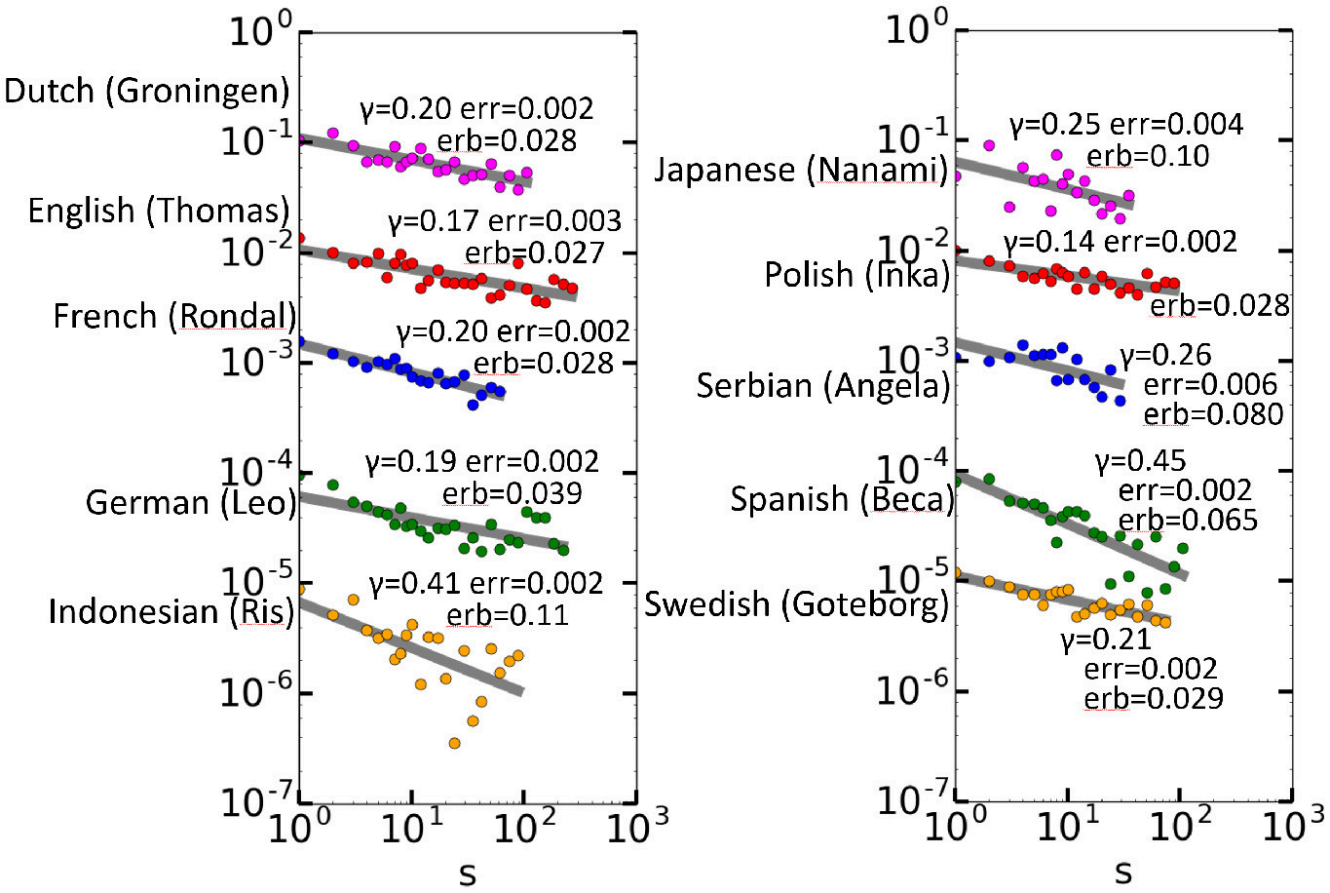
Autocorrelation functions for the 10 children. For the sake of vertical placement, the *C*(*s*) values for the *z*th data set from the top are multiplied by 1/10^*z*−1^ in each graph.

Next, the 10 longest CHILDES data sets were selected, and these included utterances in different languages (Rondal, 1985; Smoczynska, 1985; Plunkett and Strömqvist, 1992; Bol, 1995; Oshima-Takane et al., 1995; Anđelković et al., 2001; Benedet et al., 2004; Behrens, 2006; Gil and Tadmor, 2007; Lieven et al., 2009). The utterances consist of conversations between a child and adults [the mother, and in some cases, an *investigator* (a researcher)]. Every word is annotated by time and role. The data were carefully separated by speaker, and only those by children were used. Moreover, the CHILDES codes for unknown words were removed. The largest number of words was that of Thomas (in English), with 448771 words, whereas the minimum number of words was less than 48951, for Angela (in Serbian). Figure 5 shows the autocorrelation function results for Thomas and the other nine children. A tighter fit with respect to a larger data size is observed, which is deemed a statistical effect, and therefore, the power laws are not as tight for the other data as for Thomas. Nevertheless, we see that all data of 10 children are long-range correlated. Except for a single point at *s* = 10 for Ris (in Indonesian), all calculated *C*(*s*) values are positive and aligned almost linearly.

## 4. Generative language models

**Figure 6:**
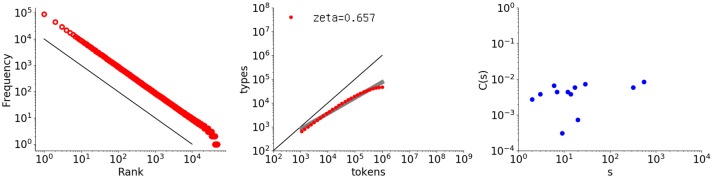
Rank-frequency distribution, type-token relation, and autocorrelation function for a randomly generated sequence that follows a Zipf distribution (1 million words with a vocabulary size of 50,000). The thick gray line shows the estimate fitting a linear model (second grpah) whereas the thin black lines indicate the slopes of –1 and 1, for the first and second graphs, respectively.

The autocorrelative characteristic reported here for children's utterances and in many previous works for natural language texts does not hold for simple random data. To demonstrate this, three examples are provided. The first example is a randomized word set whose rank-frequency sequence strictly follows a Zipf distribution^4^. Figure 6 shows graphs of the rank-frequency distribution, type-token relation, and autocorrelation function for this sequence, with a length of one million words and a vocabulary size of almost 50000 words.

The leftmost graph does exhibit a power law with the exponent −1.0, but the rightmost graph shows that the long-range correlation is completely destroyed. Many *C*(*s*) values are negative and thus not shown here because the plot is log-log. As noted before, for random data the autocorrelation function fluctuates around 0. Approximately half the values become negative and thus disappear from the figure, leaving a sparse set of plotted points, exactly as observed here.

**Figure 7:**
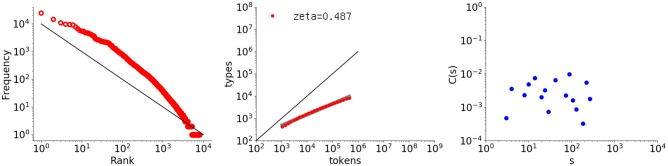
Rank-frequency distribution, type-token relation, and autocorrelation function for the Thomas data set randomly shuffled at the word level. The thick gray line shows the estimate fitting a linear model (second grpah) whereas the thin black lines indicate the slopes of –1 and 1, for the first and second graphs, respectively.

A second example was obtained by shuffling Thomas's utterances at the word level. Random shuffling destroys the original intervals between words in the Thomas data set. Figure 7 shows the analysis results, in which the autocorrelation function has become random, whereas the rank-frequency distribution and type-token relation remain the same as the original results shown in Figure 4.

**Figure 8:**
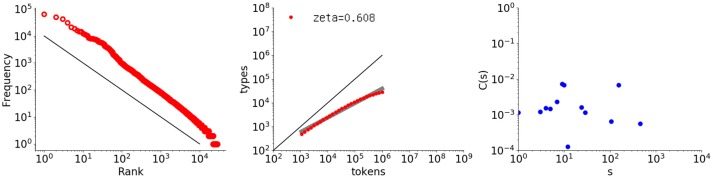
Rank-frequency distribution, type-token relation, and autocorrelation function for a sequence randomly generated from a bigram (first-order Markov) model of *Les Misérables* (one million words). The thick gray line shows the estimate fitting a linear model (second grpah) whereas the thin black lines indicate the slopes of –1 and 1, for the first and second graphs, respectively.

The third example is a Markov sequence generated using bigrams obtained from *Les Misérables*. The random sequence was generated from the bigrams according to the probabilities recorded in a word transition matrix. Figure 8 shows the analysis results, with the rightmost graph indicating that the autocorrelation function does not exhibit any memory.

Long-range correlation therefore does not hold for such simple random sequences. At the same time, given that long-range memory holds for the CHILDES data, it should be natural to consider that some simple mechanism underlies language production. In early childhood speech, utterances are still lacking in full vocabulary, ungrammatical, and full of mistakes. Therefore, the long-range correlation of such speech must be based on a simple mechanism other than linguistic features such as grammar that we generally consider.

The problem with all the findings related to power laws underlying the statistical physics domain is that even though, as mentioned before, the method has been effective for analysis in natural sciences and finance, the exact reason why such power laws hold is unknown. This applies to long-range correlation, as well: “rare words tend to cluster” is only one simplistic way to express a limited aspect of the phenomenon. As mentioned before, however, the phenomenon is more complex and has some relation to the scale-free property underlying language.

In the case of Zipf's law, Mandelbrot mathematically proved that optimizing the communication efficiency implies Zipf's law (Mandelbrot, 1952, 1965). It is unknown how this optimization theory could relate to long-range correlation. Moreover, it is not obvious whether an infant child would optimize every word of an utterance. It would be more natural to consider that a child learns how to act in choosing a word, and that this action, in fact, is mathematically bound so as to be optimal. One possible approach to understand what's behind such an *act* would be to consider the behavior of mathematical models of language with respect to power laws. Roughly, at least three representative families of mathematical processes have been considered as language models: Markov models, Poisson processes (Church and Gale, 1995) or renewal processes (Altmann et al., 2009), and recent generative models. The first two models require a predefined vocabulary size, so without further modification, they cannot be applied to confirm either Zipf's or Heaps' law. The rest of this paper therefore focuses on generative models, which naturally accommodate infinite vocabulary growth. Above all, one important aspect of these models lies in providing a model for developmental psychology (Goldwater et al., 2009, 2011).

In all the generative models presented hereafter, the model generates elements one after another, either by introducing a new word or by reusing a previous element. Let *K*_*t*_ be the number of kinds of elements (vocabulary size) at time *t*, and let *S*_*t,i*_ be the frequency of elements of kind *i* occurring until *t*. At *t* = 0, all models presented hereafter start with the following status:

$$K_{0}=1,\;S_{0,1}=1,\;S_{0,i}=0,\;i\in {\mathbb{Z}}_{>1}.$$

The most fundamental model is the Simon model (Simon, 1955) (Mitzenmacher, 2003). This model, described colloquially as “the rich get richer,” is used for a variety of natural and artificial phenomena. A similar model in complex network systems is the Barabási-Albert model (Barabasi and Albert, 1999). For *t* > 0, given a constant 0 < α < 1, an element is generated at time *t* + 1 with the following probabilities:

$$\begin{array}{l} P(K_{t+1}=K_{t}+1,S_{t+1,j}=S_{t,j},j\in {\mathbb{Z}}_{\geq 1}\backslash \{K_{t}+1\},S_{t+1,K_{t}+1}=1)\;\\ \;=\alpha ,\\ P(K_{t+1}=K_{t},S_{t+1,i}=S_{t,i}+1,S_{t+1,j}=S_{t,j},j\in {\mathbb{Z}}_{\geq 1}\backslash \{i\})\;\\ \;=(1-\alpha )\frac{S_{t,i}}{t},i=1,\;\ldots ,K_{t}. \end{array}$$

Note that the first definition gives the case when a new word is introduced, and the second gives the case when a previous word is sampled. The scheme can thus be described as follows: with constant probability α, a new, unseen element is generated; and with the remaining probability 1−α, an element that has already occurred is selected according to the frequency distribution in the past. Suppose, for example, that the previously generated sequence is *X*=[‘x', ‘y', ‘x', ‘z', ‘x', ‘z']. Then the next element will be a new element with probability α, or ‘x', ‘y', or ‘z' with probability 3(1 − α)/6, (1 − α)/6, or 2(1 − α)/6, respectively. It is trivial to understand that this reuse of previous elements is equivalent to a *uniform sampling* from the past sequence, i.e., by considering that all past elements occurred equally under a uniform distribution. In this example, uniform sampling entails picking one element randomly from *X*=[‘x', ‘y', ‘x', ‘z', ‘x', ‘z'].

It has been mathematically proven that the rank-frequency distribution of a sequence generated with the Simon model asymptotically follows a power law, independently of the value of α (Mitzenmacher, 2003). As the vocabulary introduction rate is constant, it is trivial to see that the type-token ratio also has the exponent 1.0.

To investigate the Simon model, a sequence of one million elements with α = 0.10 was generated, and its rank-frequency distribution, type-token relation, and autocorrelation function were obtained. The autocorrelation function was calculated according to the scheme explained in §2, because a new element introduced in this scheme can be anything, even a non-numerical element.

**Figure 9:**
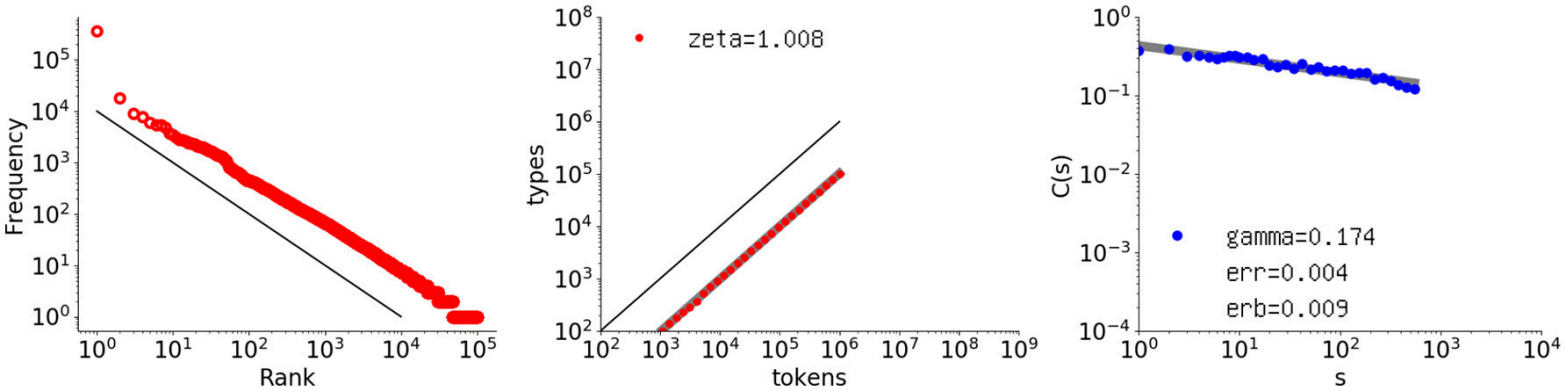
Rank-frequency distribution, type-token relation, and autocorrelation function for a one-million-element sequence generated with the Simon model, where α = 0.10. The autocorrelation function has γ = 0.174, coincidentally the same as that for Thomas, with the residual denoted as *err* and the error of the slope as *erb*. The thick gray lines show the estimates fitting a linear model (second and third graphs), whereas the thin black lines indicate the slopes of –1 and 1, for the first and second graphs, respectively.

Figure 9 shows the results. The first two graphs agree with the theory by giving exponents of −1.0 and 1.0, respectively. As for the autocorrelation function, surprisingly, long-range memory is clearly present. The slope is γ = 0.174, which is coincidentally the same as that for the Thomas data set. None of the *C*(*s*) values is negative and the fit to the slope is very tight: the slope error *erb* = 0.00895, with the fit error of *err* = 0.00369.

To examine the parameter dependence, 10 sequences for each of α = 0.1, 0.2, 0.3, 0.4 were generated, and the autocorrelation function was obtained for each. The results included no negative *C*(*s*) values. For each α, the respective mean values of γ were 0.156, 0.133, 0.118, and 0.095, with small standard deviations of 0.019, 0.018, 0.011, and 0.013, respectively. The average fit error obtained via the square error across all 40 sequences was 0.00366 per point. Thus, the slope decreased with increasing α. With large α, the Simon process has a larger number of new elements later in the sequence, and therefore, the decay of similarity between the two subsequences as measured by the autocorrelation function decreases.

The Simon model has two known problems, however, as a language model. The first is that the vocabulary growth (proven to have exponent 1.0) is too fast. Indeed, such fast vocabulary growth is very unlikely in natural language production. The second problem is that the model cannot handle the convexity underlying a rank-frequency distribution, as observed especially for the Thomas data set (Figure 4). Such convexity has been reported elsewhere, as noted before.

Another generative model called the Pitman-Yor model (Pitman, 2006) solves these two problems. Using the same mathematical notation as before, and given two constants 0 ≤ *a* < 1 and 0 ≤ *b*, the following generative process is applied for *t* > 0 at time *t* + 1 with the following probabilities:

$$\begin{array}{l} P(K_{t+1}=K_{t}+1,S_{t+1,j}=S_{t,j},j\in {\mathbb{Z}}_{\geq 1}\backslash \{K_{t}+1\},S_{t+1,K_{t}+1}=1)\\ \;=\frac{aK_{t}+b}{t+b},\\ P(K_{t+1}=K_{t},S_{t+1,i}=S_{t,i}+1,S_{t+1,j}=S_{t,j},j\in {\mathbb{Z}}_{\geq 1}\backslash \{i\})\\ \;=\frac{S_{t,i}-a}{t+b},i=1,\;\ldots ,K_{t}. \end{array}$$

As with the Simon model, the first line defines the introduction rate for new elements. It decreases with the length of the sequence, *t*, yet is linear in the vocabulary size *K*_*t*_ according to the strength *a*. This amount is generated as a sum of taking every element kind *i* = 1, …, *K*_*t*_ by subtracting *a* from frequency *S*_*t, i*_, called *discounting*, which appears in the numerator of the second definition above. Apart from this, the parameter *b* controls the convex trend (Pitman, 2006; Teh, 2006) often seen in rank-frequency distributions. When *a* = 0, this model reduces to the Chinese restaurant process (Goldwater et al., 2009), which has been applied widely in the language engineering domain.

Mathematically, the parameter *a* in the Pitman-Yor model almost equals the value of the exponent of the type-token relation, ζ, which describes the vocabulary growth speed, provided that *b* is small and Heaps' law holds (Appendix A). According to empirical verification, even for a large *b* = 10, 000, ζ only differs from *a* by a maximum of 0.1. Given this, *a* = 0.68 was chosen for the remaining Pitman-Yor models presented in this article, a value somewhat in the middle of ζ = 0.683 for the Thomas data set and ζ = 0.672 for *Les Misérables*.

**Figure 10:**
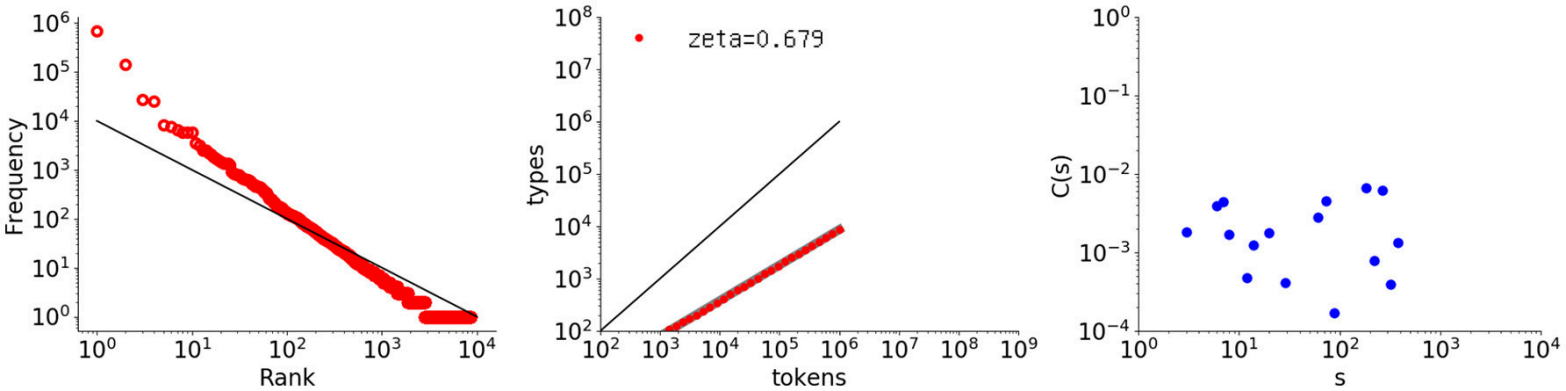
Rank-frequency distribution, type-token relation, and autocorrelation function for a sequence of one million elements generated by a Pitman-Yor model with *a* = 0.68 and *b* = 0.80. The thick gray line shows the estimate fitting a linear model (second grpah) whereas the thin black lines indicate the slopes of –1 and 1, for the first and second graphs, respectively.

For generation of one million elements by a Pitman-Yor process with *a* = 0.68 and *b* = 0.80, Figure 10 shows the three resulting graphs. Agreeing with theory, the middle graph showing the type-token relation has a slope reasonably close to 0.68. As for the leftmost graph, the rank-frequency distribution shows a power law with a slope different from –1.0. The distribution did not present a convex alignment for the sample due to *b* not being sufficiently large. In the rightmost figure, however, the power law of particular interest here, for the autocorrelation function, has disappeared. Although the change from the Simon model is subtle, with respect to the value of *a*, the sequence does not exhibit any arrangement underlying natural language. The question of why the Pitman-Yor process is not long-range correlated is difficult, because it is an extension of the Simon process with an additional parameter. It could be considered, however, that the discounting by the term *a* dramatically changes the alignment of elements within a sequence.

Because this result could be due to the parameter setting, all possible combinations of *a* = {0.0, 0.1, 0.2, …, 0.9} (10 values) and *b* = {0.0, 0.1, 0.2, …, 1.0, 10.0, 100.0, 1000.0, 10000.0} (15 values) were considered. For every pair (*a, b*) out of these 150 possibilities, a sequence of one million elements was generated and examined for long-range correlation. If any *C*(*s*) value for *s* < 10 was negative, then long-range memory was judged not to hold. This criterion is somewhat loose, because it considers long-range correlation to hold even when the points are scattered and not exhibiting power-law behavior, as long as they are still positive. Even with this loose criterion, however, none of the generated sequences has long-range correlation. When *a* is too small, the rate of introducing new words becomes too weak. Even when there are sufficient new words, the arrangement seems qualitatively different from the case of the Simon model.

We have now seen that the Simon model exhibits a bad type-token relation but a good autocorrelation, while the opposite is true for the Pitman-Yor model. Because long-range correlation is due to the arrangement of frequent words and rare words, a natural approach is to test the following conjunct generative model for *t* > 0 at time *t* + 1 with the following probabilities:

$$\begin{array}{l} P(K_{t+1}=K_{t}+1,S_{t+1,j}=S_{t,j},j\in {\mathbb{Z}}_{\geq 1}\backslash \{K_{t}+1\},S_{t+1,K_{t}+1}=1)\\ \;=\eta ,\;where\;\eta =\frac{aK_{t}+b}{t+b},\\ P(K_{t+1}=K_{t},S_{t+1,i}=S_{t,i}+1,S_{t+1,j}=S_{t,j},j\in {\mathbb{Z}}_{\geq 1}\backslash \{i\})\\ \;=(1-\eta )\frac{S_{t,i}}{t},i=1,\;\ldots ,K_{t}. \end{array}$$

This mixed model introduces new words with a probability η equal to that of the Pitman-Yor model, so the first line is exactly the same as in the definition of that model. As for sampling, with probability 1 − η a previous element is introduced in proportion to the frequencies of the elements. In other words, the conjunct model achieves uniform sampling, as in the Simon model, by replacing that model's α with η.

**Figure 11:**
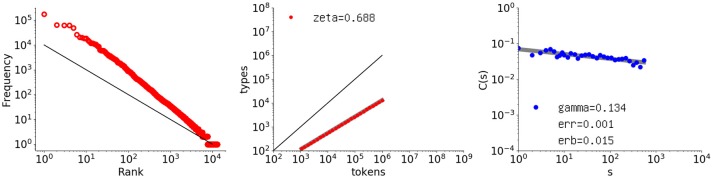
Rank-frequency distribution, type-token relation, and autocorrelation function for a sequence of one million elements generated by the proposed conjunct model with *a* = 0.68 and *b* = 0.80. The thick gray lines show the estimates fitting a linear model (second and third graphs) whereas the thin black lines indicate the slopes of –1 and 1, for the first and second graphs, respectively.

Figure 11 shows the behavior of a sequence generated by the conjunct model with *a* = 0.68 and *b* = 0.80. The model clearly exhibits the desired vocabulary growth while maintaining its long-range correlation. The exponent γ decreases to 0.134, with a slope error of *erb* = 0.0152 and residual “err = 0.00112”.

**Figure 12:**
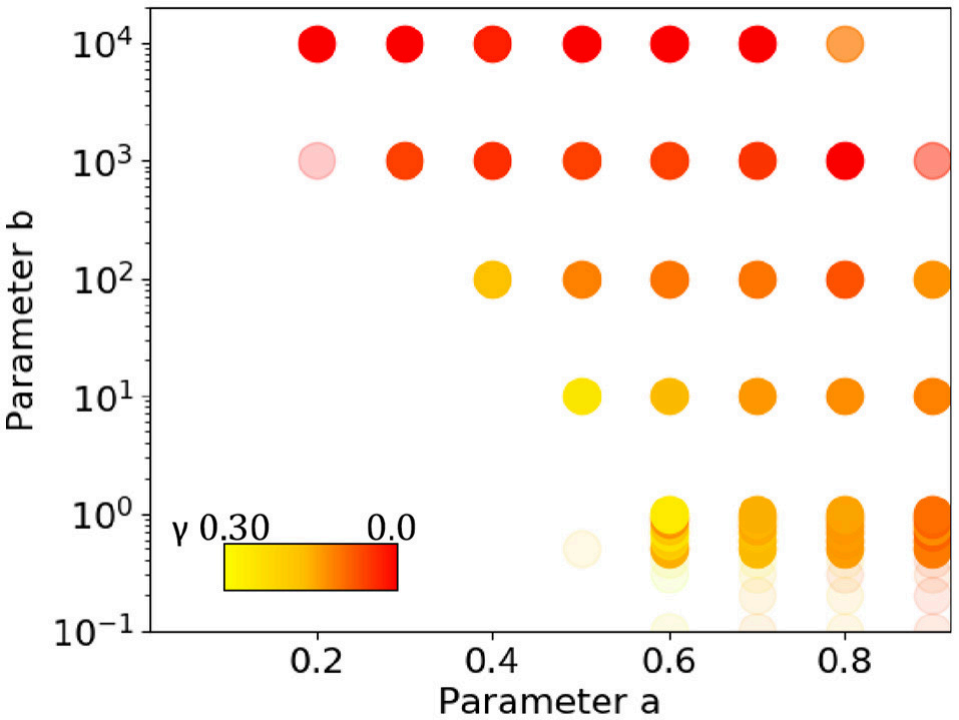
Pairs of parameters *a* and *b* for which a sequence generated by the conjunct model exhibits long-range correlation. A point is redder if γ is closer to zero, and the opacity indicates the proportion of sequences that are long-range correlated (with full opacity indicating that all samples are long-range correlated). The variance of γ is smaller when the value is larger, and more sequences are long-range correlated (more opaque and less red) toward the lower middle of the dotted area.

To examine the parameter dependence, again all possible combinations of 10 values of *a* = {0.0, 0.1, 0.2, …, 0.9} with *b* = {0.0, 0.1, 0.2, …, 0.9, 1.0, 10.0, 100.0, 1000.0, 10000.0} (15 values) were considered. For every pair (*a, b*), a sequence of one million elements was generated 10 times and examined for long-range correlation. Figure 12 shows all the pairs of values for which long-range correlation is observed. In the figure, a dot is redder when γ is close to zero but positive. The opacity indicates the proportion of samples that are long-range correlated. When *a* is too small, the rate of introducing new words becomes too small and gives no long-range correlation. For a sufficiently large *a* and a value of *b* that depends on *a*, on the other hand, long-range correlation is observed. For larger *b*, γ tends to be smaller. For other *a* values, as well, the γ values are not as high as 0.2. At the border of the area where the dots are plotted, many of the 10 samples are not long-range correlated, and the dots become more transparent. The variance of the γ values correlates with the opacity and color, being smaller toward the lower middle of the dotted area, especially for *a* = 0.6 ~ 0.9 and *b* around 1.0. The experimental setting of *a* = 0.68 and *b* = 0.80 mentioned above is in the region where the sequences exhibit long-range correlation in a stable manner. For *a* = 0.68 the average γ = 0.134, with a slope error *erb* = 0.0152 and residual *err* = 0.00112.

## 5. Discussion

The findings reported in this article lead to two main points. First, the findings raise the question of the Pitman-Yor model's validity as a language model. Pitman-Yor models have been used because they nicely model the rank-frequency distribution and the growth rate of natural language. Unlike natural language, however, the Pitman-Yor model is not long-range correlated. The fact that the Pitman-Yor process does not exhibit long-range correlation suggests that discounting the word frequency by a constant parameter *a* is questionable. In the context of developmental psychology, vocabulary acquisition cannot be explained without the capability of learning words from a few samples (Hulit and Howard, 2002; Ganger and Brent, 2004). The Pitman-Yor process does exactly the opposite by discounting constantly, putting less emphasis on fewer samples. This discounting causes large differences in the alignment of words from the Simon process, thus eliminating long-range correlation. Although the current work does not invalidate the usefulness of Pitman-Yor models for language engineering (as they are effective), the long-range correlation behavior does reveal a difference between the nature of language and the Pitman-Yor model. This could be a factor for consideration in future scientific research on language.

Second, this work reveals that among possible mathematical language models considered so far, those with uniform sampling generate strong long-range correlation (i.e., the Simon model and the conjunct model developed at the end of the previous section). Given how simple uniform sampling is, however, the findings could suggest that natural language has some connection with uniform sampling. Long-range correlation is present not only in language but also in music, as well (an example is given in Appendix B for reference), which is another human activity, similar to language. The human faculty to generate linguistic-related time series might have a fundamental structure with some relation to a very simple procedure, with uniform sampling as one possibility.

Note, however, that uniform sampling by itself is limited as a language model. In addition to the lack of linguistic grammatical features, the Simon model and its extensions exhibit different nature at the beginning and later parts of a sample: this is different from language, for which a sample from any location in the data is long-range correlated. Mathematical generative processes that satisfy all the stylized facts of language would help clarify what kind of process language is, and to this end, the proposed conjunct model could be yet another starting point toward a better language model. The conjunct model currently has two differences from actual natural language. The first is the exponent γ, which is larger for both literature and the CHILDES data, sometimes exceeding 0.3, but remains below around 0.15 for the conjunct model. Second, the rank-frequency distribution is convex for large *b* in the conjunct model, but such large *b* makes γ even smaller. Therefore, the conjunct model must be modified to address these problems. This would require more exhaustive knowledge of the nature of long-range memory in natural language.

## 6. Conclusion

This article has investigated the long-range correlation underlying the autocorrelation function with CHILDES data and generative models by using an analysis method for non-numerical time series, which was borrowed from the statistical physics domain. After first overviewing how long-range correlation phenomena have been reported for different kinds of natural language texts, they were also verified to occur for children's utterances.

To find a reason for this shared feature, we investigated three generative models: the Simon model, the Pitman-Yor model, and a conjunct model integrating both. The three models share a common scheme of introducing a new element with some probability and otherwise sampling from the previous elements. The Simon model exhibits outstanding long-range correlation, but it deviates from natural language texts by causing the vocabulary to grow too fast. In contrast, the Pitman-Yor model exhibits no long-range correlation, despite having an appropriate vocabulary growth rate. Therefore, the conjunct model uses the Pitman-Yor introduction rate for new vocabulary but samples from the past through uniform sampling, like the Simon model. This conjunct model produces long-range correlation while maintaining a growth rate similar to that of natural language text.

The fact that the Pitman-Yor model does not exhibit long-range correlation raises the question of the Pitman-Yor model's validity as a natural language model. Because the mathematical generative models of the Simon kind that exhibit long-range correlation are based on uniform sampling, we may conjecture a relation between natural language and uniform sampling. The findings in this article could provide another direction toward better future language models.

## Author contributions

The author confirms being the sole contributor of this work and approved it for publication.

### Conflict of interest statement

The author declares that the research was conducted in the absence of any commercial or financial relationships that could be construed as a potential conflict of interest.

## Figures and Tables

**Figure A1:**
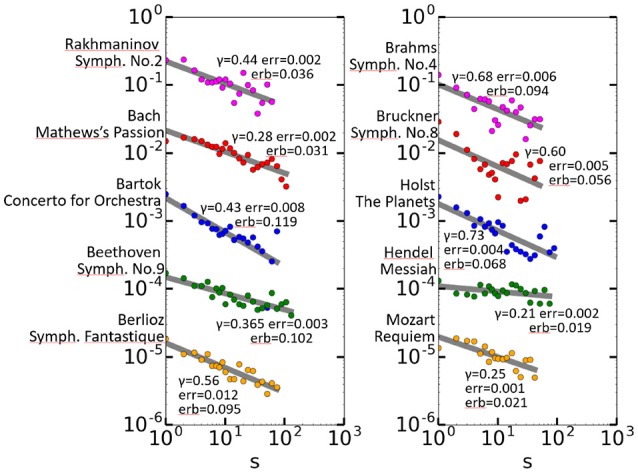
Long-range correlation of 10 long classical music pieces. For the sake of vertical placement, the C(s) values for the *z*th data set from the top are multiplied by 1/10^*z*−1^ in each graph.

## Footnotes

^1^Considering one *N*th of words as rare means that the average number of intervals is almost *N*, for any given total number of words *M*, as follows. One *N*th of words means *M*/*N* words. Then, for sufficiently large *M*, the mean interval length is (*M*−1)/(*M*/*N*−1)≈*N*.

^2^Following (Tanaka-Ishii and Bunde, 2016), the values of *s* were taken up to *M*_*N*_/100 in a logarithmic bin, which is the limit for the resulting *C*(*s*) values to remain reliable according to previous fundamental research such as that reported in Lennartz and Bunde (2009). For *s* larger than *M*_*N*_/100, the values of the points tend to decrease rapidly.

^3^The fit error reported in this article is the average distance from the fitted line for a point, or in other words, the root of all the accumulated square errors, divided by the number of points.

^4^The random sequence was generated by an inverse function method.

## Appendix A

This appendix explains why *a* ≈ ζ. At time *t*, the number of words introduced into the sequence is

(A1) $$K_{t}=\int_{0}^{t}\frac{aK_{t}+b}{t+b}.$$

Assuming that *b* is sufficiently small and $K_{t}=t^{\zeta }$,

(A2) $$\int_{0}^{t}\frac{at^{\zeta }+b}{t+b}\approx \int_{0}^{t}at^{\zeta -1}$$

(A3) $$=\frac{a}{\zeta }t^{\zeta },$$

and because this must equal *K*_*t*_, *a* = ζ. This analysis applies only to a deterministic model, whereas the model proposed in this work is stochastic. Nevertheless, empirical verification shows that *a* ≈ ζ even with stochastic simulation, provided that *b* is small.

As for the dependence on *b*, *a* ≈ ζ almost always held for *b* up to around 1.0. For larger *b*, ζ became larger than *a*: when *b* = 10, 000, for example, ζ was larger than *a* by 1.0, at most.

## Appendix B

This appendix shows the long-range correlation of 10 long classical music pieces, shown in Figure A1. The original data were performances in MIDI format, which were transformed from MIDI into text with the software SMF2MML (http://shaw.la.coocan.jp/smf2mml/). Headers and footers were eliminated so that the data contained only musical components, including pause indications. Every tune played by a different instrument kind was separated and concatenated.

For these 10 pieces the long-range correlation can be considered to hold. A previous work also reported how long-range correlation holds in skilled piano play (Ruiz et al., 2014).
